# Optimising Cancer Vaccine Design in Sarcoma

**DOI:** 10.3390/cancers11010001

**Published:** 2018-12-20

**Authors:** Alexandra Pender, Robin L. Jones, Seth Pollack

**Affiliations:** 1 Sarcoma Unit, Department of Medicine, The Royal Marsden NHS Foundation Trust, London SW3 6JJ, UK; cancers@mdpi.com (A.P.); robin.jones4@nhs.net (R.L.J.); 2 Division of Clinical Studies, The Institute of Cancer Research, London SW3 6JB, UK; 3 Fred Hutchinson Cancer Research Center, University of Washington, Seattle, WA 98109, USA; 4 Division of Oncology, University of Washington, Seattle, WA 98195, USA

**Keywords:** vaccine, sarcoma, antigen, immune response, peptide, virus, adjuvant

## Abstract

Immunotherapeutics are increasingly recognized as a key tool in the armamentarium against malignancy. The success of immune checkpoint-targeting drugs and adoptive cell therapy has refocused attention on the potential anti-cancer effect of eliciting a tumour-specific immunological response. Sarcomas are a rare and diverse group of tumours with a limited prognosis in advanced disease despite systemic therapeutics. Various vaccine strategies including peptide vaccines against cancer testis antigens, dendritic cell vaccines, and viral vectors have been trialled in sarcoma with growing evidence of efficacy. Here, we review the principles of successful vaccine development and how these have been applied thus far to the treatment of sarcoma.

## 1. Introduction

Sarcomas are a diverse group of aggressive mesenchymal tumours that represent around 1% of solid adult malignancies. Ten percent of sarcomas are bone sarcomas and 15% of sarcomas are gastrointestinal stromal tumours (GIST). GISTs are usually associated with *KIT* and *PDGFRα* mutations and are sensitive to imatinib [1]. Other common soft tissue sarcoma (STS) subtypes include leiomyosarcomas and liposarcomas. Intermediate to high grade localised sarcomas have approximately a 50% chance of relapse with 55% survival at 5 years [2]. Localised STS is managed with surgery combined with pre- or post-operative radiation. Metastatic STS has a poor prognosis of 12–18 months and is managed with systemic agents depending on histological subtype and patient performance status [3,4,5]. In-depth genomic profiling has shown that many sarcomas are generally driven by a chromosomal translocation event such as in synovial sarcoma or have copy number aberrations [6]. Some subtypes such as undifferentiated pleomorphic sarcoma (UPS) have a higher number of non-synonymous mutations and greater mutational burden [7].

Despite the origin of a malignant clone of cells from self, certain features of cancer cells mark them out as unique from the host [8]. These features, in particular expression of tumour-associated antigens, make them theoretically an attractive target for the immune system [9]. Eliciting a tumour-specific immunological response has the potential to be a significant therapeutic intervention. 

The discovery of tumour antigen specific T cells in melanoma patients with progressive metastatic disease encouraged investigation into why the immune system becomes tolerant of cancer-associated antigens [10]. Therapeutic cancer vaccines have been investigated as a potential method of stimulating an anti-cancer immune response for decades [11,12]. To date, there are two FDA-approved therapeutic cancer vaccines: sipeleucel-T in prostate cancer and intravesical bacillus Calmette-Guérin (BCG) for non-muscular invasive bladder cancer [13,14,15].

Sipeleucel-T is an autologous vaccine composed of dendritic cells exposed ex vivo to a fusion of granulocyte-macrophage colony-stimulating factor (GM-CSF) and prostatic acid phosphatase [13]. In a placebo-controlled trial of patients with castrate-resistant prostate cancer, three 2-weekly infusions of sipeleucel-T improved median overall survival (OS) by 4.1 months (hazard ratio [HR] 0.78, 95% confidence interval [CI] 0.61–0.98). Increased antibody titres to the fusion molecule and T cell proliferation responses were more frequently observed in the vaccine-treated arm than in placebo-treated patients (66.2% vs. 2.9% and 73.1% vs 12.1%, respectively). BCG is a vaccine prepared from a live attenuated strain of *Mycobacterium tuberculosis*. Intravesical BCG treatment reduces the risk of superficial bladder tumour recurrence and progression to invasive disease as compared with transurethral bladder tumour resection alone and other chemotherapeutics [15,16].

A successful cancer vaccine incorporates three key steps; selection of a tumour-specific or tumour-associated molecule, presentation of the tumour-specific molecule to the immune system, and stimulation of an adequate immune response. In this review, we will discuss how the principles of a successful vaccine have been applied thus far to the development of vaccines in the treatment of sarcomas and other strategies to alter the immune response to sarcoma which may work synergistically with vaccines.

## 2. Selection of a Tumour-Specific Molecule

A critical step in vaccine development is selection of the appropriate tumour-associated molecule to elicit an anti-tumour immune response. The ideal candidate is a molecule that is exclusively expressed by the tumour. This can encompass self-antigens, differentiation antigens specific to that cell type, abnormally or overexpressed antigens, or antigens present due to a mutated protein, also known as neoantigens [17,18,19,20] (Table 1).

Cancer testis antigens (CTAs) are germline antigens which are expressed exclusively in germ cells and in several cancer types. The expression in these cell types is 1000 fold higher than in other cells, making them good targets for cancer vaccines [21]. The absence of HLA presentation in germ cell tissue also makes these antigens tumour-specific. In addition, CTA expression is oncogenic and therefore beneficial to the tumour [22]. The first CTA identified was MAGE in melanoma, but subsequently BAGE, GAGE, SSX, LAGE-1, PRAME, and NY-ESO-1 have been identified as targets in sarcoma [23,24,25,26,27,28]. In a study screening nine different subtypes of sarcoma from 36 patients, more than 70% of tumours showed CTA overexpression [29]. PRAME, for example, is overexpressed in leiomyosarcoma and synovial sarcoma and BAGE and GAGE are overexpressed in rhabdomyosarcoma, liposarcoma, chondrosarcoma, and leiomyosarcoma [26,27,29]. NY-ESO-1 is homogenously expressed in synovial and myxoid/round cell liposarcoma [25,30]. An NY-ESO-1 peptide vaccine induced antibody and cellular immune responses in patients with minimal residual cancer that expressed NY-ESO-1 [31]. Delayed hypersensitivity (DTH) responses following vaccination correlated with survival. Upregulation of CTAs can be encouraged by demethylation using DNA methyltransferase inhibitors [32]. In ovarian cancer, this strategy has been used to augment NY-ESO-1 vaccine therapy in combination with chemotherapy with encouraging results [33]. 

Aberrant ganglioside antigen expression is also a rational tumour-specific target and vaccines have been tested in different tumour types including melanoma and sarcoma [34,35]. A bivalent peptide vaccine combining two different ganglioside antigens was trialled in stage 3 and 4 melanoma and in a synovial sarcoma patient following metastasectomy [35]. A humoral response to the vaccine was detectable in 97% of patients, but no long-term clinical data was reported. The subsequent placebo-controlled phase 2 study trialled a trivalent ganglioside vaccine in 136 patients with recurrent or metastatic sarcoma who were macroscopically disease-free after surgical metastectomy [36]. Ten doses of vaccine or placebo were given with the immunological adjuvant OPT-281 over 84 weeks. At 3 years of follow-up, the median progression-free survival (PFS) was 6.4 months with no difference between the vaccine and placebo arms, despite a sustained detectable response to vaccination in the vaccine arm. 

Unique chromosomal translocation events that are ubiquitous within a sarcoma subtype such as the t(X;18)(p11;q11) translocation in synovial sarcoma or the t(12;16)(q13;p11) in myxoid/round cell liposarcoma are attractive vaccine targets as the newly-formed peptide will potentially create a tumour-specific neoantigen [37,38]. A fragment of the SYT-SSX fusion peptide that results from the characteristic synovial sarcoma translocation was trialled as a vaccine in 21 patients with advanced synovial sarcoma [39]. One out of nine patients administered with the peptide fragment alone did not have disease progression within the study period but 6 out of 12 patients who received the peptide with an adjuvant and interferon-α had stable disease, with one short-lived clinical response. More objective measures of vaccine response were less convincing; seven patients had a greater than two-fold increase in peptide-specific circulating cytotoxic T cells but this did not correlate with clinical response and no delayed-type hypersensitivity (DTH) reactions were detectable in the participants.

Tumour cells as a whole can potentially provide a variety of tumour-associated antigens and vaccination with patient-derived tumour cells and laboratory established cancer cell lines have been attempted [20]. In one trial, 23 sarcoma patients were vaccinated with irradiated autologous tumour cells expanded from a fresh biopsy, demonstrating the feasibility of this technique [40]. Of the 16 patients available for DTH testing, 50% had a positive result after 3 weeks of vaccination. These patients had a median survival of 16.6 months versus 8.2 months for the patients who did not mount a measurable response. Vaccination with irradiated tumour cells infected by a replication-deficient adenovirus encoding granulocyte-macrophage colony stimulating factor (GM-CSF) can encourage tumour immune cell infiltration and necrosis [41]. Tumour cell cultures from metastatic lesions were infected with adenovirus encoding human GM-CSF, irradiated, and aliquoted into vaccines in one study. Eleven patients with metastatic clear cell sarcoma and alveolar soft part sarcoma who had received at least one prior therapy received a course of at least three autologous tumour cell vaccinations (maximum 13). Antibody responses to angiopoietin 1 and 2, upregulated tumour-associated antigens, were measurable in 9 of the 10 patients that could be evaluated. Strong cellular infiltrates at the injection site and positive DTH tests to irradiated non-infected tumour cells were detectable by the fifth vaccination. At week 10, seven of the 10 patients who received at least six vaccinations had stable disease on imaging. This may imply efficacy but may also reflects the indolent clinical course of these subtypes of sarcoma and a small patient cohort [42]. 

## 3. Presentation of the Tumour-Specific Molecule to the Immune System

To elicit a host cellular immune response, a tumour-associated antigen is presented by major histocompatibility (MHC) molecules [43]. One method of tumour immune evasion is MHC downregulation through loss of expression on the tumour cell surface. Gene expression for HLA-A, B, and C as well as TAP1, which plays a role in peptide loading onto MHC class 1, is higher in UPS and leiomyosarcoma as compared with liposarcoma and synovial sarcoma [7,44]. Downregulation of MHC class 1 expression has been observed in other series of bone and STS [45,46] and is linked to poorer prognosis in Ewing sarcoma [47]. 

Direct delivery of antigen-presenting cells within the vaccine can be a method of presenting the tumour-associated antigen to the immune system. Autologous dendritic cells that have been exposed to tumour-associated antigens or whole tumour cell lysates are strategies that have been trialled in sarcoma [48,49,50,51]. Sixteen patients with bulky Ewing sarcoma or rhabdomyosarcoma were apheresed and the immature dendritic cells were then exposed repeatedly to peptides developed from the fusion proteins seen in these sarcomas [6,48]. Intravenous vaccination of the exposed dendritic cells was given alongside continual infusion of interleukin-2 (IL_2). One patient in the cohort showed a mixed clinical response and another showed evidence of a proliferative immune cell response specific to the fusion peptide. A similar approach was used to treat two children included in a Japanese dendritic cell vaccine study, one with Ewing sarcoma and one with synovial sarcoma [49]. Immature dendritic cells were pulsed with tumour-specific peptides designed to include the junction region of the EWS-FLI-1 and SYT-SSX2 fusion proteins seen in these sarcoma sub-types and predicted to have a high affinity for the patient’s HLAA2+. Exposure to tumour peptides was also in the presence of a marker immunogenic antigen keyhole limpet hemocyanin (KLH), recombinant GM-CSF, and interleukin-4. Vaccines were administered after the patients had completed conventional multi-modality treatment followed by an autologous stem cell transplant and had evidence of disease progression or residual tumour. Specific T cell responses were detectable in both patients following the vaccination course. The child with Ewing sarcoma had a complete remission following therapy which was sustained after 77 months of follow-up. This patient also had a detectable increase in NK cell cytotoxic activity for 12 months after completion of the vaccine course. Autologous dendritic cells exposed to breakpoint peptides specific to alveolar rhabdomyosarcoma or Ewing sarcoma were delivered alongside autologous lymphocytes, an influenza vaccine and recombinant IL_2 to 30 patients who had completed standard multimodality therapy [50]. An influenza vaccine was given as an internal control alongside the tumour-specific vaccine as prior studies have suggested that certain T cell subsets are slow to recover from cytotoxic chemotherapy and response to vaccines may therefore be suboptimal [51]. A sustained response to the influenza vaccine was measurable in all patients despite prior dose-dense chemotherapy. Thirty-nine percent of patients showed an initial immune response to the tumour-specific peptide but a sustained response was not detectable at 6 months. Immune response to the tumour peptide did not correlate with survival. Fifty-two patients were initially enrolled and apheresed prior to commencing multi-modality treatment and only 30 went on to receive immunotherapy due to patient choice (10/52), progressive disease (11/52) or death (1/52). There was an improvement in five-year OS in the patients that received immunotherapy post multimodality therapy (43%) in comparison with all patients and those who received multi-modality therapy alone (31% and 15% respectively). This should be interpreted with caution as 12 of the 22 patients not treated with immunotherapy were not eligible to receive it due to progressive disease or death [50]. 

Dendritic cell vaccines exposed to tumour-associated antigens have been combined with other therapeutics in sarcoma. In an early phase trial, 10 children with relapsed cancer including one with Ewing sarcoma and one with rhabdomyosarcoma received four cycles of the hypomethylating agent decitabine, followed by two vaccinations with dendritic cells exposed to MAGE-A1, MAGE-A3, and NY-ESO-1 peptides [52]. The rationale for using decatibine was to upregulate antigen expression on the tumour cells [53]. A CD4+ T cell response was measurable in the sarcoma patients after the vaccine but not a CD8+ T cell response. Both sarcoma patients progressed during therapy but patients with neuroblastoma treated in the same study had more encouraging clinical responses [54]. 

Maturing dendritic cells in the presence of tumour cell lysate can expose the dendritic cell to a greater number of tumour-associated antigens. A phase 1 study trialled a vaccine of autologous immature dendritic cells pulsed with irradiated tumour lysate from the patient and KLH [55]. One child with metastatic fibrosarcoma had a partial response and had serological evidence of a response to tumour lysate. A larger trial in high-risk paediatric sarcoma, including patients with Ewing sarcoma, rhabdomyosarcoma, synovial sarcoma, desmoplastic small round cell sarcoma, and undifferentiated sarcoma, recruited 29 patients who were treated with vaccine following multi-modality treatment [56]. Autologous leucocytes were treated to deplete regulatory T cells and any tumour cells present [57]. Dendritic cells were divided, pulsed with the patient’s tumour lysate or KLH, and then recombined. The vaccine was administered with recombinant IL-7 to encourage lymphocyte repopulation and an infusion of treated leucocytes was given during vaccine treatment [58]. Five-year PFS and OS was better in the patients with Ewing sarcoma or rhabdomyosarcoma (40% vs. 0% and 63% vs. 0% respectively). Seventy-one percent of Ewing sarcoma and rhabdomyosarcoma patients had no evidence of residual disease after multimodality treatment prior to immunotherapy, as compared with 12.5% of patients with other sarcoma subtypes. Overall there was a non-significant trend towards improved survival with immunotherapy. All patients who received three vaccines had a T cell response to KLH and a greater proportion of patients had an immune response to tumour lysate after vaccination than before (58% vs. 31%). Patients with an immune response had an improved five-year OS (*p* = 0.0172) [56].

Delivery of the antigen presenting cell directly into the tumour could induce a tumour-specific immune response. The combination of radiation and tumour-specific vaccines has been shown to increase anti-tumour immune responses in an animal model, possibly due to the upregulation of Fas ligand on tumour cells [59]. Tumoural dendritic cell injection has been trialled alongside neoadjuvant radiation in STS [60,61]. Seventeen patients with high risk (> 5 cm) localized sarcomas including synovial sarcoma, undifferentiated pleomorphic sarcoma, fibrosarcoma, epithelioid sarcoma, MPNST, and myxoid/round cell liposarcoma received three weekly intratumoural injections of dendritic cells during a 28-day course of radiation. The dendritic cells extracted from each patient prior to radiation were infected with adenovirus expressing survivin during maturation. Following vaccine treatment, 53% patients demonstrated an immune response to either tumour cell lysate or survivin. Immune responses to survivin were longer-lasting that those observed with tumour lysate. Patients with an immune response had significantly higher levels of CD4^+^ T cells infiltrating the tumour and non-responders had a higher level of myeloid derived suppressor cells. Overall clinical outcomes were similar to that expected in this population with a 12-month disease free survival of 70.6%. A second phase 1 trial studied the combination of radiation with intratumoural dendritic cell injection in a similar population [61]. Eighteen patients with intermediate to high grade STS larger than 5 cm were enrolled to receive 4-weekly autologous dendritic cell vaccines during a course of 50 Gy of radiation delivered in 25 fractions. The last dendritic cell vaccine was given after completion of radiotherapy and before surgical resection. Ten out of 18 patients had UPS, 2/18 had epithelioid sarcomas, and 2/18 had sarcomas with myxoid features. As per the previous study, dendritic cells were infected with an adenovirus expression survivin before vaccine administration. Ten out of 18 (56%) patients demonstrated an immune response to tumour cell lysate or survivin at one point following treatment. There was a trend towards improved survival in the responders compared with the non-responders. After a median follow-up of 4.4 years, the median two-year survival was 83%. Eight patients showed a disease-free interval of 6–8 years of which five were responders to therapy. 

Another mechanism of antigen delivery is using recombinant viral vectors expressing the tumour-associated antigen which are taken up by dendritic cells [62]. This approach can elicit a stronger anti-tumour cellular and humoral response than a peptide vaccine [63]. Recombinant fowlpox and vaccinia vaccines expressing NY-ESO-1 have been trialled in sarcoma patients with high tumour NY-ESO-1 expression [64]. Twenty-three patients with advanced solid tumours, including four sarcoma patients, received at least four vaccinations at 4 weekly intervals. Twenty of 23 patients developed either a humoral or cellular response to NY-ESO-1 and CD8+ T cells from these patients were able to recognize native NY-ESO-1 in vitro in contrast to previous peptide vaccination studies [31]. Similar responses were seen with either fowlpox or vaccinia vectors [64]. Three patients with measurable metastatic sarcoma at enrolment had disease progression on trial, one of whom did not mount an anti-tumour immune response. Patients in the trial with metastatic melanoma had some subjective clinical benefit. 

A modified integration-deficient lentivirus vaccine encoding full-length NY-ESO-1, LV305, has a Sindbis virus envelope which binds DC-SIGN cell surface markers specific to immature dendritic cells [65]. Subcutaneous injection lends itself to this approach due to the number of dendritic cells in the skin and these have been demonstrated to then elicit a cellular immune response [66]. LV305 has shown considerable activity in sarcoma [67]. The vaccine elicits a dose-dependent response to challenge by metastatic lung cancer expressing NY-ESO-1 in animal models [68]. The phase 1 study of LV305 included 23 evaluable sarcoma patients. Seventy percent developed a NY-ESO-1 -specific cellular response [69]. Clinical benefit was seen in 58% sarcoma patients, with one late partial response and five patients with benefit for over a year. One patient with treatment-refractory recurrent NY-ESO-1 positive synovial sarcoma received three doses of LV305 and developed a strong NY-ESO-1-specific T cell response. Her disease subsequently responded and the response has been maintained for over 2 years since vaccination [67].

Dendritic cells can also be targeted using the DEC-205 receptor. Antigen-antibody conjugates result in durable antigen-specific cellular responses and this approach has been incorporated into NY-ESO-1 vaccine development [70,71].

## 4. Stimulation of an Adequate Immune Response

Prior vaccine studies have shown that presentation of a tumour neoantigen can often only lead to a minimal immune response [62]. Adjuvant signals or molecules can make this response more robust and durable by recruiting a greater immune response. This has the benefit of reducing the amount of vaccine required and increasing the speed of the immune response [72]. Theoretically the addition of radiation or chemotherapy will cause more tumour cell death and release of further tumour-associated epitopes. A second tumour-associated peptide or a known immunogenic peptide can be injected simultaneously or at a later time-point. Aluminium salts (alum) have been used for many years to recruit immune cells and increase antigen uptake, although alum does not stimulate a CD8^+^ T cell response [73]. MN59 is an oil-in water adjuvant used in the influenza vaccine which activates cytokines and stimulates chemokine production but does not elicit a cellular immune response [74]. Saponin-based adjuvants (plant-derived glycosides) have been used in a NY-ESO-1 peptide vaccine amongst others [31,75]. Toll-like receptor (TLR) agonists, including imiquimod, have been used in several vaccines, including in sarcoma [20]. TLR agonists, such as monophosphoryl lipid A (MPA), potently activate dendritic cells and CD4+ cells [76]. Combinations of adjuvants e.g., alum and MPA, are in use in licensed vaccines and further combinations are in development [72].

Using different types of vaccine with the same tumour-associated antigen also improves the immune response. This strategy has been successful in synovial sarcoma and myxoid liposarcoma with a CMB305 vaccine regimen targeting NY-ESO-1 [43]. The host is primed with four LV305 vaccinations carrying the tumour antigen, alternating with the G305 vaccine-carrying tumour antigen and the TLR4 agonist, glycopyranosyl lipid A, which is a potent dendritic cell activator. The first G305 injection is given after two LV305 vaccines and continues 4-weekly until the LV305 course is completed, after which it is given 8-weekly for up to a year. Forty-nine patients, including 25 pre-treated sarcoma patients, were treated with the CMB305 vaccine regimen in a phase 1 clinical trial [77]. All had NY-ESO-1 positive disease and 70% developed anti-NY-ESO-1 antibodies. The median progression-free-survival was 4.7 months and 76% patients were alive 18 months post vaccination. A patient who had an extreme response within the early phase 1 study (ongoing response at 3 years) had T cell recognition of NY-ESO-1 at baseline and an increase in the percentage of NY-ESO-1 positive T cells expressing PD-1. The presence of either antibodies or a T cell response to NY-ESO-1 before or during treatment correlated with a prolonged PFS and OS. Patients with the most prolonged PFS and OS had a baseline immune response that was increased following vaccination; i.e., a greater than fourfold antibody response or a greater than twofold T cell response [43]. 

A different vaccine strategy with other adjuvants also focuses on the NY-ESO-1 antigen in sarcomas [70]. The CDX-1401 vaccine comprises the NY-ESO-1 antigen fused to DEC-205 (CD205), the endocytic dendritic cell receptor. On binding the dendritic cell, NY-ESO-1 is then delivered directly for antigen presentation. Five sarcoma patients were treated within a 45-patient phase 1 clinical trial of this vaccine. CDX-1401 was given twice a week for up to four doses over a six-week period. Resiquimod, a TLR 7/8 agonist or Hiltonol, a TLR3 agonist were also administered alongside CDX-1401. Forty-one patients had at least one six-week cycle of treatment and ten patients went on to further treatment. 79% of patients had measurable NY-ESO-1 antibodies post vaccination, although 54% of patients with NY-ESO-1 positive tumours had detectable antibodies at baseline. Fifty-six percent of patients had detectable antigen-specific T cell responses and this was associated with stable disease after vaccination. Four NY-ESO-1 positive melanoma and two non-small cell lung cancer patients who participated in the Phase 1 study went on to receive checkpoint inhibitors within 3 months of vaccination and had a partial or complete response to treatment. 

The tumour microenvironment is critical to a strong immune response after vaccination. Removing inhibitory signals at the immune checkpoint may play a key role in successful vaccination. Immunosuppression within the tumour microenvironment, characterised by lymphocyte depletion, macrophage infiltration and TGF-B signalling, correlates with worse prognosis across tumour types [78]. Tumours with high lymphocytic infiltrates are either associated with a high proliferation index and heterogeneity, escape from immune recognition (immune edited tumours) and poorer prognosis or low proliferation, low heterogeneity, immune equilibrium, and better prognosis. Somatic mutations in driver oncogenes may also affect the immune response. Some of these features have been observed in sarcoma [7,79]. UPS, the subtype with the highest mutational burden, has a more robust T cell infiltrate, high PD-1 and PD-L1 expression and high expression of genes related to antigen presentation [7]. High PD-L1 expression in alveolar soft part sarcoma may contribute to immunosuppression and the limited response observed to vaccine therapy despite the presence of a T cell infiltrate [79].

Direct therapeutic approaches to release an immune response are the administration of immune checkpoint inhibitors such as CTLA-4, PD-1, and PD-L1 inhibitors and adoptive cell therapies. An 86 patient single arm phase 2 trial of pembrolizumab in the seven commonest forms of bone and STS showed activity in UPS and dedifferentiated liposarcoma [80]. Patients who had received up to three lines of prior systemic treatment could be enrolled in the study. Of the 80 patients in whom response was evaluable, 7/40 (18%) STS patients had a partial response to treatment. Overall, the responses were durable with a median duration of response of 33 weeks (95% CI 23–49). The median PFS for all STS patients was 18 weeks (95% CI 8–21) which increased to 30 weeks (95% CI 8–68) and 25 weeks (95% CI 8–42) for UPS and liposarcoma, respectively. One patient with UPS had a complete response of 13 months duration. Median OS for the STS cohort was 49 weeks (95% CI 34–73) as compared with an OS that was not reached in the UPS patient cohort after a median of 19.1 months of follow-up. PD-L1 expression was seen in two STS samples, both from patients with UPS who responded to treatment. Liposarcoma patients who responded had PD-L1 negative tumours. Following this study, expansion cohorts are currently in progress for pembrolizumab in UPS and poorly differentiated liposarcoma. The PEMBROSARC study assessed efficacy of pembrolizumab with metronomic cyclophosphamide in metastatic sarcoma using pre-defined tumour subtype-specific cohorts [81]. Of the 50 patients assessed for response, no patients in the UPS or liposarcoma cohorts in this study responded to pembrolizumab. One patient with a solitary fibrous tumour with PD-L1 expression on >10% tumour cells had a response to treatment. A high proportion of tumours from patients enrolled in this study had a heavy macrophage infiltration, possibly suggesting an immune suppressed phenotype. 

Single-agent nivolumab and combination immunotherapy with ipilimumab and nivolumab have been trialled in metastatic sarcoma patients recruited to parallel phase 2 trials [82]. The response rate to nivolumab monotherapy was 5% (92% CI 1–16) as compared with 16% (95% CI 7–30) of patients treated with combination immunotherapy. Responses on the combination treatment were in patients with leiomyosarcoma, UPS/malignant fibrous histiocytoma, myxofibrosarcoma, and angiosarcoma. 

Adoptive cell therapy is currently being investigated in many tumour types including sarcoma [83]. T cells engineered to express a modified high affinity T cell receptor targeted to NY-ESO-1 have shown efficacy in metastatic treatment-refractory sarcoma [84,85]. The majority of a cohort of 18 sarcoma patients had frequent and durable responses and the estimated three-year survival was 38%. A further study treated 12 treatment-refractory metastatic synovial sarcoma patients with genetically engineered NY-ESO-1 specific T cells [86]. The response rate was 50% (6/12) and the median duration of response was 30.9 weeks (range 13–72). 

The addition of vaccines to immune checkpoint therapies may be a potent combination. Following the promising phase 1 trial results with the CMB305 vaccine and the observation that an extreme responder to the vaccine had a greater percentage of PD-1 positive NY-ESO-1 positive T cells, the CMB305 regimen has been combined with atezolizumab in a randomised 88-patient phase 2 study in locally advanced or metastatic NY-ESO-1 positive sarcoma [77,87]. The interim results for 36 patients with 7 months of follow-up showed an improved median PFS in the combination arm of 2.6 months over 1.4 months with atezolizumab alone [87]. More mature results are awaited but this combination of immunotherapeutics appears to augment the response to either agent alone and may influence survival. 53% of patients in the vaccine plus atezolizumab arm demonstrated an NY-ESO-1 specific cellular response and 41% had a specific antibody response as compared with 25% and 0% for the atezolizumab arm, respectively. 

## 5. Conclusions

The clinical outlook of locally advanced and metastatic sarcoma is limited with a median overall survival, even with salvage second-or third line therapies, in the order of 12–18 months [5,88,89]. The development of cancer vaccines has included various approaches in patients with sarcoma with little observed toxicity. The identification of a ubiquitous tumour-specific antigen NY-ESO-1, an efficient delivery mechanism, an effective dosing regimen, and a potent adjuvant has resulted in a potentially efficacious adjunct to the therapeutic repertoire. A phase 3 clinical trial of maintenance CMB305 following first-line chemotherapy in advanced synovial sarcoma is currently closed to recruitment pending initial results and more mature data are awaited for the combination of atezolizumab and CMB305. A trial combining CMB305-, LV305-, and NY-ESO-1-specific adoptive T cell therapy is currently recruiting. Dendritic cell vaccine trials are also ongoing in sarcoma. Despite the previously observed low response rate to cancer vaccines [62], the vaccine story in sarcoma continues and suggests that there is a place for this approach in cancer care in the 21st century. 

## Figures and Tables

**Table 1:** Summary of vaccine trials in sarcoma using various tumour-specific target molecules.

Tumour-Specific Molecule	Example in Sarcoma	Population	Intervention	*n*	Outcomes	Reference
Differentiation antigens	NY-ESO-1 peptide vaccine	Minimal residual disease following resection of NY-ESO-1 positive tumours	Three doses of peptide vaccine +/−adjuvant at 4 week intervals; various dose levels of peptide and adjuvant	46	All patients who received vaccine with an adjuvant had a measurable serological response 10/16 patients who received vaccine and adjuvant had a DTH response NY-ESO-1 specific T cell responses detected in all patients tested	[31]
Ganglioside antigens	Trivalent ganglioside conjugate vaccine	Recurrent or metastatic sarcoma, macroscopically disease-free post metastectomy	10 doses of vaccine or placebo given with OPT-281 over 84 weeks	136	PFS 6.4 months in vaccine and placebo arms	[36]
Neoantigen secondary to chromosomal translocation event	SYT-SSX fusion peptide	Advanced synovial sarcoma	9 patients received peptide vaccine alone; 12 patients received peptide vaccine + adjuvant + interferon-α Six doses were given at 14 day intervals	21	Peptide vaccine alone: 1/9 stable disease Peptide vaccine + adjuvant + interferon-α: 6/12 stable disease	[39]
Autologous tumour cells	Expanded tumour cells from fresh biopsy, irradiated	Recurrent or metastatic sarcoma	Vaccine weekly for 3 weeks and then monthly for up to 5 months (total 6 months or 8 doses)	23	Median survival of 16.6 months for patients who demonstrated positive delayed hypersensitivity three weeks post vaccination versus 8.2 months	[40]

Key: *n*, number of patients; DTH, delayed-type hypersensitivity; PFS, progression-free survival.
